# The adaptation of educational expectations in response to ability tracking: Variations by migration background

**DOI:** 10.1111/1468-4446.12886

**Published:** 2021-08-17

**Authors:** Sara Geven, Andrea G. Forster

**Affiliations:** ^1^ Department of Sociology University of Amsterdam Amsterdam the Netherlands; ^2^ Department of Education and Psychology Freie Universität Berlin Berlin Germany

**Keywords:** ability tracking, education, expectations, inequality, migration background

## Abstract

In various educational systems, students are sorted into separate secondary schools on the basis of their academic ability. Research suggests that this type of tracking impacts students' educational expectations, as expectations generally align with students' ability track. However, most research is cross‐sectional and students with lower expectations are possibly also sorted into lower tracks. Moreover, the extent to which track placement influences expectations may vary across students. In this paper, we address the following research question: how does ability tracking impact the development of student expectations and how does this vary by students' migration background. Based on the literature on the immigrant aspiration–achievement paradox, we expect that students with a migration background are less likely to adapt expectations downwardly, and more likely to adapt expectations upward in response to track placement. Using German panel data, we examine the educational expectations of students with and without a migration background, before and after track placement. Moreover, we use variations in tracking procedures across German states to study how students who get tracked compare with students who do not get tracked in the development of their educational expectations. We show that students are more likely to upwardly adjust their expectations when their track placement exceeds their expectations and to downwardly adjust their expectations when their track placement is below their expectations. We find little support for the hypothesized variations by student migration background. Students whose parent(s) hold (a) Bachelor degree(s) are more likely to upwardly adjust their expectations when their track placement exceeds their expectations than students whose parent(s) maximally hold an upper secondary or vocational degree(s).

## 1 INTRODUCTION

Having high expectations is often considered to be a key to success in the educational context. Although unrealistic educational expectations can lead to disappointments, research shows that (somewhat) optimistic expectations generally translate into better educational outcomes, including higher levels of school motivation, effort (Domina et al., 2011), achievement, and attainment (Beal & Crockett, 2010; Pinquart & Ebeling, 2020).

Because of the positive educational consequences associated with high educational expectations, it is crucial to understand their origins in order to inform policies intending to improve educational attainment and potential inequalities herein. Scholars suggest that educational expectations are not only a consequence of individual dispositions or a student's family environment but are also shaped by the institutional context such as the educational system in which a student is embedded (e.g., Finger, 2016; Kerckhoff, 1977). One important aspect of the institutional context for the development of expectations is the level of between‐school ability tracking (Buchmann & Dalton, 2002; Buchmann & Park, 2009). Between‐school ability tracking, also termed external differentiation, is a key feature of the level of stratification of the educational system and refers to the formal tracking of students into different schools according to their academic performance level (Heisig & Solga, 2015). Students in different schools/tracks follow different curricula and are prepared for different educational trajectories (e.g., university or vocationally oriented programs) (Van de Werfhorst & Mijs, 2010). While various countries are characterized by comprehensive schooling systems (e.g., Norway, Spain, the United Kingdom, the United States), between‐school ability tracking is ubiquitous in multiple European countries (e.g., Germany, Austria, Czech Republic, Luxembourg, the Netherlands, Switzerland) (Buchmann & Dalton, 2002; Heisig & Solga, 2015).

Research shows that in countries that are characterized by a high level of between‐school ability tracking—so‐called highly differentiated systems—students' expectations tend to align with the track that they attend (Buchmann & Park, 2009). In comparison to students attending vocationally oriented tracks, students attending academic tracks are far more likely to expect to obtain a college degree or more. Moreover, in more differentiated systems, the educational attitudes of parents and peers are less strongly related to a student's educational expectations (Buchmann & Dalton, 2002). Possibly, this is due to the fact that school tracks send such a clear message to students about their educational future options that they override the influence of significant others.

Unfortunately, existing studies on the relationship between ability tracking and expectations are primarily cross‐sectional, making it impossible to ascertain whether expectations are formed prior to, or as a consequence of, track placement. An exception to this is a study by Karlson (2015): Using longitudinal data, he shows that American students adapt their expectations to the track in which they are placed in high school. However, American students are tracked on a course‐by‐course basis, and tracking may shape students' daily experiences and expectations even more in countries in which students are tracked into entirely different schools for their full curriculum (c.f. Domina et al., 2017; Kerckhoff, 1977).

While educational tracking seems to channel educational expectations, these tracking effects may not be uniform for all students. Scholars have warned against an essentialist take on students' responses to tracking, as there are large differences between students within the same track (Gamoran & Berends, 1987; Geven, 2019). In this paper, we follow up on this by arguing that tracking effects on expectations may vary between students of the native majority, Western migration backgrounds, and non‐Western migration backgrounds. According to the literature on the immigrant aspiration–achievement paradox, students with a migration background generally tend to perform less well in school compared with their native counterparts, yet, given this, they tend to have relatively high educational aspirations and expectations (Friberg, 2019; Hadjar & Scharf, 2018; Heath et al., 2008; Kao & Tienda, 1995; Salikutluk, 2016). We argue that this discrepancy may be due to the fact that students with a migration background are less responsive to educational performance signals including track placement.

We examine variations by students' migration background in how student expectations for high school graduation develop in the period before and after track placement in Germany. Germany presents an ideal case to study the impact of ability tracking on expectations, as Germany is characterized by a stringent form of between‐school ability tracking, yet federal states also vary in their tracking procedures. While, in most states, students are placed into ability tracks for their full curriculum after Grade 4, in some states, tracking occurs after Grade 6. This allows us to compare changes in the educational expectations of students who are placed in a specific track to changes in the expectations of students *in the same country* who are not yet tracked. Previous studies on the role of ability tracking in educational expectations have often relied on cross‐country comparisons (e.g., Buchmann & Dalton, 2002; Buchmann & Park, 2009). Our within‐country comparison has advantages over a cross‐country approach, as various potentially confounding factors can be kept constant (e.g., nationwide policies and institutions, the structure of the labor market, the main migration backgrounds of the student population). While we focus on a sole (between‐school tracking) country, we discuss how our findings can be informative for other settings.

## 2 THEORY

### 2.1 Tracking and educational expectations

Research on educational expectations has often relied on the Wisconsin Model of Status Attainment (Sewell et al., 1969, 2003). According to this conceptual model, educational expectations are shaped by a student's academic performance as well as significant others—parents, peers, and teachers—who are also affected by a student's academic performance. Educational expectations are also hypothesized to be socially stratified: students from advantaged social backgrounds would not only perform better in school but would also receive more positive evaluations, expectations, and encouragement from significant others.

The Wisconsin model has been criticized for being too individually focused and for ignoring institutional constraints and opportunity structures (Finger, 2016; Kerckhoff, 1977; Sewell et al., 2003). Institutional constraints and opportunity structures—including the constraints imposed by tracking institutions—are believed to especially affect educational expectations (i.e., *realistic* aspirations) rather than *idealistic* aspirations (Finger, 2016). Idealistic aspirations refer to students' educational wishes, whereas realistic aspirations or expectations refer to educational intentions or plans.

One important institutional factor that is believed to influence students' educational expectations is the level of between‐school ability tracking or educational differentiation (Buchmann & Dalton, 2002; Buchmann & Park, 2009; Kerckhoff, 1977). Educational differentiation refers to the extent to which students are sorted into different educational programs on the basis of their academic performance from an early age (Buchmann & Park, 2009; Van de Werfhorst & Mijs, 2010). In highly differentiated systems, students from different programs (i.e., tracks) follow entirely different curricula and are prepared for different educational trajectories. Typically, only students in the highest academic track can directly enter higher education. Moreover, the tracking system tends to be rigid: once students attend a specific track, upward mobility is difficult.

Various scholars also hypothesize that students will receive stronger signals about their educational performance and opportunities in more strongly differentiated systems, causing them to be more realistic about their future educational outcomes (Buchmann & Dalton, 2002; Buchmann & Park, 2009; Kerckhoff, 1977). Alternatively, track placements may influence student expectations because they come to serve as a self‐fulfilling prophecy (de Boer et al., 2010). Track placements could signal to students an educational expectation. Even if this expectation is unfounded, students may internalize the expectation, such that it *becomes* reality. More specifically, students' academic self‐concept and expectations may be negatively affected by the allocation to a relatively “low” track and positively affected by the allocation to a relatively “high” track.

A few empirical findings indeed suggest that students may adapt their expectations to their track level. More specifically, cross‐sectional research shows that students' realistic expectations and their track level tend to align (Buchmann & Park, 2009). Relatedly, studies show that students adapt their expectations to new information about their academic performance (Andrew & Hauser, 2011; Karlson, 2019) including their track placement in specific courses (Karlson, 2015). However, adaptations in expectations tend to be modest and only occur when there are large shocks in performance information (Andrew & Hauser, 2011). In any case, students seem to primarily adapt their educational expectations when the new information does not align with their previous expectations. Hence, we hypothesize:

Hypothesis 1a Students who are placed in a track that exceeds their initial educational expectations are more likely to adapt their expectations upwardly than (1) students whose track placements matches their initial expectations, (2) students whose track placement is below their initial expectations, or (3) students who are not yet placed in a specific track.

Hypothesis 1b Students who are placed in a track that is below their initial educational expectations are more likely to adapt their expectations downwardly than (1) students whose track placements matches their initial expectations, (2) students whose track placement exceeds their expectations, or (3) students who are not yet placed in a specific track.

### 2.2 Variations by a student's migration background

While theoretical accounts and empirical findings suggest that educational expectations tend to align with students' academic performance, studies have noted important differences in this alignment by a student's migration background. Empirical findings show that children from immigrant parents generally perform worse in school than their native majority peers in terms of grades and test scores, yet—given this lower performance level—they have relatively high expectations (Engzell, 2019; Heath et al., 2008; Kao & Tienda, 1995; Salikutluk, 2016). This advantage, also referred to as “immigrant optimism” or “the immigrant aspiration–achievement paradox,” is visible with respect to idealistic aspirations as well as realistic ones (Friberg, 2019; Hadjar & Scharf, 2018).

In the literature, four possible theoretical explanations have been put forward for this relative asymmetry between the educational performance and expectations among students with a migration background (Engzell, 2019; Jackson et al., 2012; Kao & Tienda, 1995; Salikutluk, 2016). First, in their decision to migrate, immigrant parents may have been partly motivated by a wish or expectation to have a more prosperous life in the destination country, and they socialize their children with this ambition (Kao & Tienda, 1995; Salikutluk, 2016).

Second, lacking information about the educational system in the destination country, immigrant parents, and their children may develop unrealistically high expectations as they are unable to judge (the consequences of) academic performance signals (Salikutluk, 2016).

Third, immigrant children may aim for higher education degrees, as they foresee discrimination in the labor market, and the attainment of a higher education degree may be perceived as a protection against discrimination (Jackson et al., 2012). The additional value that students with a migration background attach to educational qualifications may also reduce the perceived costs of continuing in education and thereby could enhance educational ambitions.

Finally, migrants tend to be positively selected, implying that in the context of their country of origin, their educational level is relatively high (Engzell, 2019; Feliciano & Lanuza, 2017). Because of this, their culture tends to correspond with that of parents from advantaged socioeconomic backgrounds: They expect or desire that their children will achieve a relatively high social status in the destination country. However, in absolute terms, and in context of the destination country, the educational level of immigrant parents tends to be relatively low (Engzell, 2019). This could cause children from immigrant parents to be equipped with relatively high aspirations and expectations, whereas simultaneously having fewer skills and resources to perform well in school in the context of the destination country.

The mechanisms that are thought to be responsible for the relatively high expectations among students with a migration background may also cause them to respond differently to new information about their educational performance, including being placed in a lower or higher ability track than expected. First, students from immigrant backgrounds may generally respond less to new information about their educational performance than students from the native majority. Due to a lack of knowledge about the destination country's educational system, immigrant parents may be more likely to stick with their original expectations when being confronted with new information about their child's performance. Language problems may inhibit immigrant parents to become (fully) informed about new information about their child's school performance (Bonizzoni et al., 2016). Moreover, even if they do receive this information, they may be less well equipped to adequately interpret it. For example, they may not know that only the academic track grants access to university. Given that parental expectations are important in shaping student expectations (Sewell et al., 2003), it is likely that this also leads to greater persistence in educational expectations among children of immigrants.

Hypothesis 2a Compared to students from the native majority, students from migration backgrounds are less likely to adapt their expectations downwardly or upwardly when they are, respectively, placed in a track that is below or above their initial educational expectations.

However, theoretically, it is also possible that students from immigrant backgrounds are less likely to adapt their expectations when being confronted with *negative* information about their academic performance, yet are more likely to adapt their expectations when receiving *positive* performance information. The high motivations and ambition for upward mobility, and the desire to circumvent discrimination in the labor market, may cause students with a migration background to keep on pursuing high expectations even in the light of academic setbacks (c.f. Geven, 2019). Moreover, given that migrants tend to be positively selected, their responses to new information about their child's academic performance may resemble that of parents from advantaged social backgrounds. That is, due to their strong desire for the status maintenance, they are expected to be more likely to stick to high expectations no matter what constraints they are facing (c.f., Breen & Goldthorpe, 1997; Finger, 2016; Forster, 2021). Conversely, their desire for status maintenance and high ambitions may cause them to more quickly increase their expectations in light of new positive information about their academic performance.

Hypothesis 2b Compared to students from the native majority, students from immigrant backgrounds are more likely to adapt their expectations upwardly when being placed in a track that exceeds their expectations, yet less likely to adapt their expectations downwardly when being placed in a track that is below their expectations.

Some empirical findings are in line with the theoretical expectation that students with a migration background are less likely to adjust their expectations to new (negative) information about their academic performance. More specifically, a meta‐study finds weaker associations between early academic achievement and later educational expectations in studies that include a higher share of minority students (Pinquart & Ebeling, 2020). Relatedly, a literature review on racial differences in student motivations shows that, compared to White students, African‐American students are more likely to keep on expecting to do well in future tasks in the light of current task failures (Graham, 1994). Contrary to this, Karlson (2019) finds no ethnic differences in the relationship between educational performance and expectations in the United States.

While the abovementioned studies shed light on the impact of negative performance signals on educational expectations, they do not explicitly focus on the impact of tracking signals. Most research on between‐school tracking has studied the impact of between‐school tracking on inequality in student performance or attainment, showing that in systems in which students are tracked at a younger age into different schools, there are larger disparities in educational performance and attainment by socioeconomic and migration background (Griga & Hadjar, 2014; Van de Werfhorst & Mijs, 2010). So far, no studies have examined differences by migration background in the impact of tracking on educational expectations. However, a study using data on students in England, the Netherlands, and Sweden shows that students of the native majority in lower ability tracks tend to decrease their commitment to school more over time, and this does not apply to students with a migration background (Geven, 2019).

All in all, the relatively high ambitions among students with a migration background may be (partly) due to the fact that these students respond less to new negative information about their academic performance and more to positive information. However, based on current studies on the immigrant aspiration–achievement paradox, we cannot infer this, as most studies are cross‐sectional and do not focus on how the development in performance is related to changes in expectations (e.g., Friberg, 2019; Hadjar & Scharf, 2018; Salikutluk, 2016). Relatedly, there may be other reasons for the relative misalignment. For example, discriminatory practices in school may cause children from migration backgrounds to receive lower grades and track recommendations than their native counterparts, even if their academic ability is the same (Glock & Krolak‐Schwerdt, 2013; Klapproth et al., 2018). This may in turn cause them to hold higher expectations than equally performing but less able counterparts from the native majority. Hence, to investigate whether students with a migration background respond differently to new information, a longitudinal design is warranted to establish how the availability of new information (i.e., track placement) is related to *alterations* in the educational expectations of both the native majority and the children with a migration background.

### 2.3 The German context

The German educational system is characterized by a high level of differentiation. In addition, the tracking system does vary to a certain extent across federal states. In most federal states, children are placed in separate ability tracks already after fourth grade (at the age of 10). In two states (Berlin and Brandenburg), track placement happens only after sixth grade. We use this difference in our analysis to compare students who are tracked to those who are (not yet) tracked. Furthermore, there are some differences across federal states in how many tracks are offered, what the exact requirements are for attending a particular track and how the process of track placement is organized. However, overall, external differentiation is strong in all federal states (der Kultusministerkonferenz, 2015). In some federal states, comprehensive schools (Gesamtschule) do exist which offer all tracks under one roof, however, they play a rather minor role in the educational system.

There is some permeability between the different tracks, but upward transitions from lower to higher tracks are not common (Jacob & Tieben, 2009). Only the highest ability track (Gymnasium) prepares students for directly entering university after obtaining an Abitur‐diploma, whereas the intermediate (Realschule) and lower ability tracks (Hauptschule) lead toward vocational training programs or colleges.

Next to differentiation, other features of the German educational system might influence student expectations. While in other countries, for example, the United States, college expectations have become the norm (Goyette, 2008), the German case is more nuanced. On the one hand, German higher education does not involve high direct costs as most universities are public and do not charge tuition fees. Additionally, public loans and stipends are available to low‐income students which make higher education accessible to students of all backgrounds. On the other hand, viable alternatives to higher education exist in the strong vocational training sector. A substantial part of each student cohort enters apprenticeships that are well acknowledged on the labor market. They have the potential to divert especially students from lower social backgrounds away from more prestigious higher education (Shavit & Muller, 1998). While student expectations are also on the rise in Germany, they might still be more strongly differentiated than in other contexts.

These institutional structures might interact with the migration background of students. The main immigrant groups in Germany come from Turkey, countries of the Former Soviet Union (FSU), former Yugoslavia, as well as Eastern European countries (especially Poland and Romania). Many immigrants with Turkish backgrounds are descendants of former guest workers of lower socioeconomic status who migrated to Germany in the 1960s and 1970s. Migrants from the FSU countries are typically ethnic Germans who resettled in Germany after the end of the Soviet Union in the early 1990s and have a diverse socioeconomic status. In the early 1990s, many refugees and asylum seekers from former Yugoslavia entered Germany. In more recent years, a larger number of labor migrants entered Germany from Eastern Europe especially since the integration of many Eastern European countries into the European Union. However, many of these workers only stay in Germany temporarily (Ohliger, 2008). Finally, in recent years, also the number of immigrants with Middle Eastern backgrounds has been growing many of whom came to Germany as refugees (Statistisches Bundesamt, 2019). Due to their recent arrival, integration processes are still very much ongoing and the socioeconomic standing of these groups in Germany is mostly low.

Students from all these groups might be subjected to the immigrant aspiration–achievement paradox as several of the mentioned mechanisms apply to them. Some of these groups, such as migrants from Turkey or FSU countries, have largely migrated for economic reasons and might, therefore, have high wishes for a prosperous life in Germany (Castles & Miller, 2009). Furthermore, while minorities stemming from the Middle East, North Africa, or Pakistan experience most discrimination in the German labor market and also minorities destining from culturally or geographically nearer‐by countries (e.g., Russia) are confronted with discrimination (Di Stasio & Bram, 2020; Koopmans et al., 2019) or may expect to be discriminated against. Experimental research indicates that cultural and geographical distance plays a larger role than racism in labor market discrimination in Germany (Di Stasio & Bram, 2020; Koopmans et al., 2019). In the German labor market, discrimination can already enter in hiring processes for firm‐based apprenticeships, making vocational trajectories less viable for immigrant students (Schneider et al., 2014). Hence, they might develop high expectations to take the route via tertiary education into the labor market.

For the current study, we focus on the distinction between students of the native majority, students with a non‐Western migration background, and students with a Western migration background, as (most of) the mechanisms that are supposed to underlie the aspiration–achievement paradox are hypothesized to apply to all students with a migration background, yet arguably more to students originating from non‐Western countries. However, as student experiences may be dependent on a student's specific migration or ethnic background, we also perform robustness checks in which we consider differences between (1) students from the native majority, (2) students with a Turkish background, (3) students with an FSU or Polish background, and (3) students with another migration background.

## 3 DATA

We use data from the National Educational Panel Study (NEPS): Starting Cohort Kindergarten, doi:10.5157/NEPS:SC2:8.0.0 (Blossfeld et al., 2011). From 2008 to 2013, NEPS data were collected as part of the Framework Program for the Promotion of Empirical Educational Research funded by the German Federal Ministry of Education and Research (BMBF). As of 2014, NEPS is carried out by the Leibniz Institute for Educational Trajectories (LIfBi) at the University of Bamberg in cooperation with a nationwide network.

Children were followed yearly from preschool (age 4, year 2010–2011) into secondary school (2017–2019; sixth grade; data collection still ongoing). A two‐stage sampling design was used to select a nationally representative starting sample of all German children attending preschool. First, a size‐proportional random sample of German primary schools was drawn. Subsequently, a random sample of all preschools that were feeding into these primary schools was drawn. All 4‐year old children from the sampled preschools were invited to participate. In the third year, the entire first grade of the selected primary schools was invited to participate. Children who did not enter a sampled primary school were invited to participate individually. This led to a sample of 9,336 children.

Student expectations were measured from third grade onward. In all federal states except for Berlin and Brandenburg, students attend a tracked school in their fifth grade of primary school (age 10). In Berlin and Brandenburg, children are selected into a track in their seventh grade.

Since we examine differences in expectation before and after track placement, we only keep students who participated both in third (before track placement) and in fifth grade (just after track placement, except for students in Berlin and Brandenburg). We also drop students who repeated a grade, as they may make the transition at a different time point. This leaves us with a sample of 2,771 students.

We impute missing values on the independent variables by means of multiple imputations using chained equations in Stata 16. For student characteristics, we impute 20 data sets, using all (student level) predictor variables in the analyses, the dependent variables (i.e., educational expectations), and auxiliary variables (e.g., students' idealistic aspirations, school effort, math ability, reading ability, and gender; parental realistic and idealistic aspirations, help in school, unemployment status, and occupational status; teacher track expectations; federal states). We impute variables in a wide format so that missing values on one observation point can be imputed by nonmissing values on another observation point. We do not impute the dependent variable, as this can lead to noise in the estimates (Young & Johnson, 2015).

## 4 MEASUREMENTS

To measure *expectations*, we use a question in which students were asked which high school‐leaving qualification they were likely to obtain, considering their current knowledge. In Waves 7 and 8, when most students were in secondary school, there were three answer options: a leaving qualification of the Hauptschule, Realschule, or Abitur. In Waves 5 and 6, students could also indicate that they thought they would leave school without a qualification. Answers were recoded into a dichotomous measure that indicated whether or not students expected to obtain the leaving certificate of the academic track (Abitur). We use this dichotomous measure, as the tracks that are offered vary across German states, yet the academic track (Gymnasium) is consistently offered in all states (Forster, 2021). Table 1 shows that student expectations are relatively high. On average, 64% of the students expects to finish the academic track in third grade, and this increases to 73% in sixth grade. Descriptively, we do not observe clear variations in (the development of) student expectations by migration background.

**TABLE 1:** Descriptive statistics

	Native majority	Non‐Western migration background	Western migration background	Total
Expects academic track third grade	0.64	0.66	0.65	0.64
Expects academic track fourth grade	0.71	0.72	0.73	0.71
Expects academic track fifth grade	0.71	0.70	0.75	0.72
Expects academic track sixth grade	0.72	0.76	0.77	0.73
Match expectation track				
Expectation = track	0.55	0.57	0.58	0.55
Expectation > track	0.06	0.08	0.08	0.06
Expectation < track	0.14	0.15	0.16	0.14
Not tracked	0.25	0.20	0.18	0.24
Track placement				
Nonacademic track	0.16	0.18	0.18	0.17
Academic track	0.59	0.61	0.64	0.60
Not tracked	0.24	0.20	0.18	0.24
Parental education	7.57	6.05	6.26	7.32
≤Inter. Sec.	0.01	0.20	0.17	0.04
Upper Sec., Voc	0.49	0.41	0.51	0.49
≥Bachelor	0.50	0.39	0.32	0.47
*N*	2,258.4	156.9	355.7	2,771

Note

Data: NEPS SC2, version 8.0.1, own calculations. Shares and numbers of observations are averaged over 20 imputed data sets.

To measure the match between students' initial expectations and track placement, we construct a categorical variable with four categories. The first category (*expectation* *= track*) refers to students who expected to obtain the Abitur in third grade and were also placed in the academic track (Gymnasium) or who expected to obtain a qualification from a nonacademic track in third grade and were also placed in a nonacademic track (Realschule or Hauptschule). The second category refers to students whose track placement exceeded their expectations. That is, they expected to receive a qualification from a nonacademic track in third grade, yet attended the academic track in Grade 5 (*expectation* *< track*). The third category refers to students whose expectations exceeded their track placements (*expectation* *> track*): They expected to receive an academic track qualification in third grade, yet attend a nonacademic track in fifth grade. The last category consists of students who are still in primary school/attend a school with multiple tracks^1^ (*not tracked*). Note that this last category only includes students in specific regions (i.e., students living in federal states where tracking occurs later or where students can attend combined tracks). By distinguishing between students who are sorted into specific tracks and students who are not yet tracked, we thus take into account the different tracking systems in the different federal states.^2^

We use parental country of birth to identify a student's migration background. We distinguish between students of the native majority, students with a non‐Western migration background, and students with a Western migration background.^3^ Students whose parents originate from European countries and countries where the dominant language is English are considered to be of Western descent (cf., Kruse, 2017). Students whose parents originate from FSU countries are considered to have a Western background, while students whose parents originate from Turkey are considered to have a non‐Western background. If one parent is native‐born, whereas the other parent is a migrant, we rely on the country of birth of the migrant parent. When both parents are migrants, yet originate from a different country, we rely on the mother's country of birth. Children with German‐born parents who are born abroad are considered to be from the native majority.

Table 1 shows the number of students in each of the migration background groups, averaged across the imputed data sets. About 2,258 students are considered to have a native majority background, 157 students have a non‐Western background, and 356 students have a Western background.

Since student experiences may depend on their specific ethnic or migration background, we also perform robustness checks in which we distinguish between (1) students from the native majority, (2) students with a Turkish background, (3) students with a background from the FSU or Poland, and (4) students with another migration background. Unfortunately, the number of students with a migration background in the data does not allow us to make more fine‐grained distinctions than this.

In some of the models, we account for how changes in expectations vary by a student's socioeconomic status (SES). We measure SES with a categorical variable indicating the educational attainment of the biological parent with the highest educational qualifications. We distinguish between (1) intermediate secondary school‐leaving qualification or less (ISCED < 3), (2) upper secondary school‐leaving qualification, apprenticeship, or vocational qualification (ISCED 3‐5B), and (3) a Bachelor degree or more (ISCED 5A, 6). To construct this variable, we use the ISCED‐97 scores in the NEPS data set that are based on information provided by one of the parents/caregivers.^4^ If the educational attainment of one of the parents was missing, we rely on the information of the parent whose information is not missing. When the educational attainment of both biological parents is missing, we use the educational attainment of the caregiver who is primarily responsible for the care of the child or the educational level of the (partner of the) caregiver who lives in the same home as the child. Table 1 shows that parental education is typically lower for students with a migration background than for students of the native majority.

## 5 ANALYTICAL APPROACH

To estimate how to track placement is related to alterations in students' educational expectations, we use a difference‐in‐difference (DiD) design. We estimate linear probability models (LPMs) to estimate our dichotomous outcome variable (students' expectation to obtain the Abitur), as LPMs are more straightforward to interpret than logistic regression models and allow for a more forthright estimation of interaction effects (Ai & Norton, 2003). More specifically, we analyze how to track placement (i.e., the treatment) is related to changes in students' likelihood to expect the Abitur before (third grade) and after track placement (fifth grade). Here, track placement refers to whether a student's Grade 5 track placement matches, exceeds, or is below a student's Grade 3 expectations. Our final model can be summarized by the following equation:

$$\begin{array}{c} y_{\mathrm{it}}=\beta_{0}+\beta_{1}\text{Time}\;+\beta_{2}\text{Time}*\text{Track Placement}\\ \left(+\beta_{3}\text{Time}*\text{Migration Background}+\beta_{4}\text{Time}*\text{Track Placement*Migration Background}\right)\\ \left(+\beta_{5}\text{Time}*\text{SES}+\beta_{6}\text{Time}*\text{Track Placement}*\text{SES}\right)\\ +\mu_{i}+\varepsilon_{\mathrm{it}}, \end{array}$$

where $y_{\mathrm{it}}$represents the likelihood to expect to obtain the Abitur of person *i* at time point *t*, $\mu_{i}$represents the individual fixed effects, and $\varepsilon_{\mathrm{it}}$represents the error term. Tracking effects (*β*_2_) are estimated by interacting time with the variable indicating whether students' track placement matches initial expectations. Here “time” refers to the grade that students are in. Subsequently, we study how these tracking effects vary by a student's migration background by including a three‐way interaction between time, the track placement variable, and student migration background (*β*_4_).^5^ In this model, we also account for the underlying two‐way interaction between time and migration background (*β*_3_). Finally, we estimate models in which we also account for a three‐way interaction between time, track placement, and student SES (*β*_6_). Students from migration backgrounds tend to be from disadvantaged socioeconomic backgrounds, and this may cause them to hold lower expectations and to be more responsive to negative performance signals and less responsive to positive performance signals (Forster, 2021). This may suppress the hypothesized migration background effects. In this model, we account for the two‐way interaction between time and student SES (*β*_5_).

The DiD design accounts for all individual‐level variables that are constant over time, including time‐invariant differences in students' cognitive competencies/academic ability/academic performance. Nevertheless, it should be noted that the estimates may be confounded by individual‐level variables that change over time. Since students move to a new school, it is possible that students experience changes that are unrelated to their track placement that *does* influence their expectations.

We account for the nesting of students in primary schools by adjusting the standard errors for this clustering. All estimates we present are unstandardized coefficients.

## 6 RESULTS

Table 2 shows the results of the DiD models predicting changes in students' likelihood to expect to obtain the Abitur (i.e., academic track school‐leaving certificate) between third and fifth grade. The findings are based on a sample of 5,542 observations nested in 2,771 students.

**TABLE 2:** DiD model predicting changes in student expectations

	M1	M2	M3
Grade (ref. = Grade 3)			
Grade 5	−0.001	−0.002	−0.002
	(0.007)	(0.008)	(0.013)
Grade * Match expectation–track (ref. = Expectation = track)			
Grade 5 * Expectation > track	−0.717***	−0.752***	−0.776***
	(0.033)	(0.036)	(0.045)
Grade 5 * Expectation < track	0.887***	0.904***	0.854***
	(0.018)	(0.019)	(0.031)
Grade 5 * Not tracked	−0.016	−0.016	−0.110**
	(0.028)	(0.030)	(0.037)
Grade * Migration background (ref. = Native majority)			
Grade 5 * Non‐Western		0.034	0.032
		(0.038)	(0.039)
Grade 5 * Western		−0.004	−0.006
		(0.023)	(0.025)
Grade * Match expectation–track * Migration background			
Grade 5 * Expectation > track * Non‐Western		−0.023	−0.055
		(0.136)	(0.173)
Grade 5 * Expectation > track * Western		0.228*	0.212
		(0.112)	(0.122)
Grade 5 * Expectation < track * Non‐Western		−0.193*	−0.157
		(0.094)	(0.085)
Grade 5 * Expectation < track * Western		−0.040	−0.001
		(0.055)	(0.060)
Grade 5 * Not tracked * Non‐Western		−0.167	−0.121
		(0.147)	(0.153)
Grade 5 * Not tracked * Western		0.087	0.133
		(0.093)	(0.089)
Grade * Parental education (ref. = Upper Sec., Voc)			
Grade 5 * ≤ Inter. Sec.			0.014
			(0.060)
Grade 5 * ≥ Bachelor			−0.001
			(0.015)
Grade * Match expectation–track * Parental education			
Grade 5 * Expectation > track * ≤ Inter. Sec.			0.064
			(0.193)
Grade 5 * Expectation > track * ≥ Bachelor			0.076
			(0.080)
Grade 5 * Expectation < track * ≤ Inter. Sec.			−0.089
			(0.123)
Grade 5 * Expectation < track * ≥ Bachelor			0.090*
			(0.038)
Grade 5 * Not tracked * ≤ Inter. Sec.			−0.090
			(0.162)
Grade 5 * Not tracked * ≥ Bachelor			0.255***
			(0.059)
Constant	0.641***	0.641***	0.641***
	(0.004)	(0.004)	(0.004)
*N*	5,542	5,542	5,542

Note

Data: NEPS SC2, version 8.0.1. Estimates are averaged over 20 imputed data sets. Standard errors in parentheses. All models contain individual‐level fixed effects.

* *p* <.05

** *p* <.01

*** *p* <.001.

### 6.1 Tracking and educational expectations

In the first model, we test Hypotheses 1a and 1b and analyze how the (mis)match between a student's initial expectations and track placement is related to *changes* in the likelihood to expect the Abitur. This is estimated by means of an interaction between (1) the grade a student is in and (2) the (mis)match between a student's track and initial expectations.

In Model 1, we find no main effect of “grade.” This implies that students whose track in fifth grade aligns with their Grade 3 expectations (the reference group) do not alter their Abitur expectations between third and fifth grades. The change in Abitur expectations for students who are not tracked does not differ from that of students whose fifth‐grade track placement aligns with their initial expectations (i.e., there is no statistically significant interaction between grade and students who are not tracked). Average marginal effect calculations show that the likelihood to expect the Abitur also does not change between third and fifth grades for students who are not tracked.^6^

We find a positive interaction between grade and students whose track placement exceeds their initial expectations, and a negative interaction effect between grade and students whose track placement is below their initial expectations. In line with Hypothesis 1, this implies that the likelihood to expect the Abitur increases more for students who are placed in a track that exceeds their expectations than for students whose track placement matches their expectations (Hypothesis 1a‐1). The likelihood to expect the Abitur increases less for students who are placed on a track below their expectations than for students whose track placements match their expectations (Hypothesis 1b‐1).

Average marginal effect calculations reveal that the likelihood to expect the Abitur increases between third and fifth grade for students whose track placement exceeds their initial expectations, while it decreases for students who are placed in a track that is below their initial expectations. In line with our hypotheses, the difference between these two groups is statistically significant (Hypotheses 1a‐2 and 1b‐2), and they also statistically significantly differ from students who are not tracked after fourth grade (and who experience no statistically significant changes in their Abitur expectations) (Hypotheses 1a‐3 and 1b‐3).

### 6.2 Variations by a student's migration background

In Model 2, we test Hypotheses 2a and 2b. That is, we study variations by students' migration background in the relationship between the (mis)match in initial educational expectations and track placement on the one hand and changes in students' likelihood to expect the Abitur on the other hand.

In line with Hypothesis 2a and in contrast to Hypothesis 2b, we find that among students whose track placement exceeds their initial expectations, the likelihood to expect the Abitur increases less for students with a non‐Western migration background than for students of the native majority (i.e., the interaction between grade, non‐Western migration background, and having expectations that are below track placement is negative and statistically significant). We find no statistically significant interaction for students with a Western migration background.

In line with Hypotheses 2a and 2b, we find that among students whose track placement is below their initial expectations, the likelihood to expect the Abitur decreases less for students with a Western migration background than for students of the native majority (i.e., there is a statistically significant negative interaction between grade, having expectations that exceed track placement, and Western migration background). In contrast to Hypotheses 2a and 2b, no such interaction is found for students with a non‐Western migration background.

We find no statistically significant differences by migration background in changes in Abitur expectations among students who are not tracked and students whose track placement meets their prior expectations. Average marginal effects calculations reveal that, irrespective of a student's migration background, these students do not significantly alter their likelihood to expect the Abitur over time.

**FIGURE 1:**
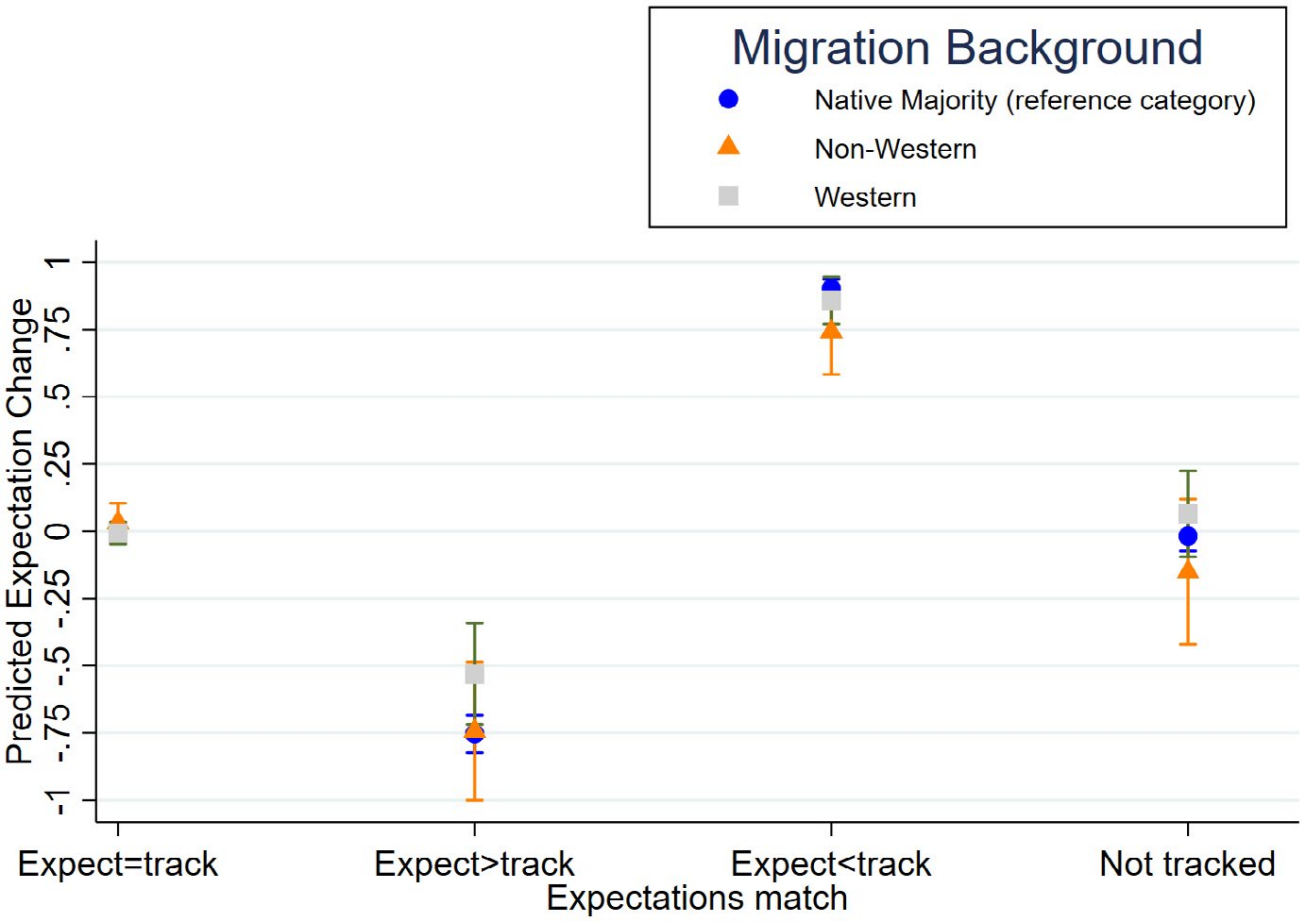
Average marginal effects of being placed in a track that matches or does not match prior expectations by migration background. Calculations are based on the estimates of Model 3 in Table 2

So far, we have found little support for Hypothesis 2a or Hypothesis 2b. Possibly this is due to the fact that students' migration background is conflated with their socioeconomic background. Children of migrant parents might be from lower socioeconomic backgrounds than children from the native majority, and this SES background may cause them to be (1) less likely to upwardly adjust their Abitur expectations when their track placement exceeds their initial expectations and (2) more likely to downwardly adjust their Abitur expectations when their track placement is below their initial expectations (cf., Forster, 2021). Hence, in Model 3, we account for interactions between parental education, the (mis)match between initial expectations and track placement, and grade. Figure 1 displays the average marginal effects of experiencing a (mis)match between initial expectations and track placement and changes in the likelihood to expect Abitur between third and fifth grade for the different migration background groups after accounting for the interactions with parental education.

In contrast to the findings from Model 2, Model 3 shows no statistically significant interactions between migration background, grade, and experiencing a (mis)match between initial expectations and track placement (Table 2 and Figure 1). The fact that we do not find a statistically significant interaction anymore between non‐Western migration background, grade, and being placed in a track that exceeds initial expectations may be due to the fact that students from non‐Western migration backgrounds tend to be from lower socioeconomic backgrounds than native majority students. More specifically, findings show that among students who are placed in a track that exceeds their initial expectations, the likelihood to expect the Abitur increases less for students whose parents maximally hold an upper secondary or vocational degree than for students whose parent(s) hold (a) Bachelor degree(s).

**FIGURE 2:**
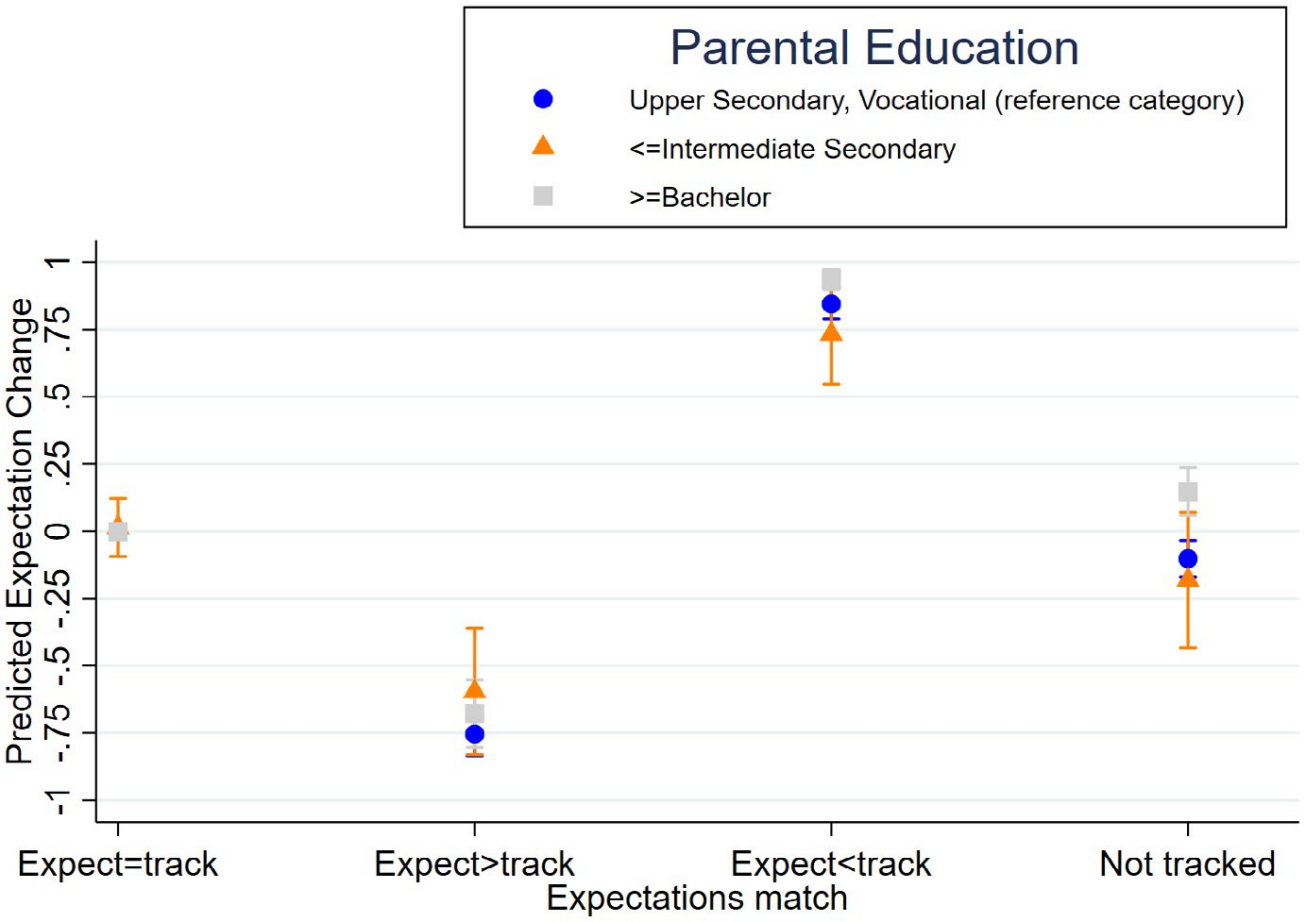
Average marginal effects of being placed in a track that matches or does not match prior expectations by parental education. Calculations are based on the estimates of Model 3 in Table 2

Figure 2 displays the average marginal effects of experiencing a (mis)match between initial expectations and track placement by the three parental education groups. Aside from the fact that students whose parent(s) hold (a) Bachelor degree(s) increase their likelihood to expect the Abitur more when their track placement exceeds their initial expectations than students whose parents maximally hold an upper secondary or vocational degree, we also find variations by parental education among students who are not tracked in fifth grade. Students whose parent(s) hold (a) Bachelor degree(s) increase their likelihood to expect the Abitur over time, while students whose parents hold lower educational certificates decrease or do not alter this expectation over time. Possibly students who are not tracked already start to receive “track‐related” or “achievement‐related” signals in fifth grade (e.g., teachers' track placement expectations), which may explain this difference by parental SES.

### 6.3 Additional analyses

We conduct several additional analyses. First, we estimate models in which we distinguish between students of the native majority, students with a Turkish background, students with an FSU/polish background, and students with another migration background. The findings of these models are presented in Table A1. Again, we find no statistically significant differences by students' migration background in how a (mis)match in initial educational expectations and track placement is related to changes in the likelihood to expect to obtain the Abitur.

Second, we perform analyses in which we examine how placement into a *specific track* is related to *yearly* changes in Abitur expectations between third and sixth grades, and how this varies by a student's migration background. These findings are based on a sample of 10,236 observations nested in 2,771 students. We distinguish between students who have placed in a nonacademic track (Realschule or Hauptschule), the academic track (Gymnasium), or not yet tracked (i.e., in primary school/attending a school with multiple tracks). Table A2 (Appendix) presents the findings of these models.

In Model 1, we analyze the relationship between track placement and changes in Abitur expectations for all students, not testing for variations by students' migration background. We find that, after track placement, students who are placed in the nonacademic track (reference group) decrease their likelihood to expect the Abitur (i.e., negative main effects for Grades 5 and 6). Moreover, we find positive and statistically significant interaction effects between (1) students placed in the academic track and the different grades and (2) students who are not tracked and Grades 5 and 6. Average marginal effect calculations reveal that students in the academic track experience a statistically significant increase in their likelihood to expect the Abitur, while students who are not tracked do not alter this expectation over time.

The Abitur expectations of students placed in a nonacademic track start to diverge from those of students who are not tracked *after* track placement takes place. However, already *before* track placement takes place (between third and fourth grade), students who get placed in the academic track increase their Abitur expectations more than students who get placed in the nonacademic track or students who are not tracked. Hence, these differences are not (only) due to track *placement* itself. Nevertheless, it is important to note that students already receive “tracking” signals/labels before actual track placement, as students who are tracked in fifth grade receive a track recommendation in fourth grade. Findings do show that students who are placed in the academic track experience an additional rise in their Abitur expectations right after track placement.

In Models 2 and 3, we test for variations by migration background. In Model 2, we do not account for the fact that changes in expectations vary by parental education, whereas in Model 3, we do. Both models indicate that the relationship between track placement and changes in the likelihood to expect the Abitur do not vary by students' migration background.

## 7 CONCLUSION

In this paper, we examined how students adapt their expectations in response to their track placement and how these adaptations varied by a student's migration background. We used German panel data that enabled us to study the educational expectations of students before and after track placement and compare these to the expectations of students who were not yet placed in an ability track. In line with previous cross‐sectional research (Buchmann & Dalton, 2002; Buchmann & Park, 2009), we found that students align their expectations with their ability track: the likelihood to expect the Abitur (i.e., academic track school‐leaving certificate) increased among students whose track placement exceeded their initial expectations, yet decreased among students whose track placement was below their initial expectations. For students who were placed in a nonacademic track, we found a drop in the likelihood to expect the Abitur right after track placement. Such a drop was not visible among students who were not tracked. Similar to how student expectations tend to be influenced by signals about academic performance (Andrew & Hauser, 2011; Karlson, 2019), students seem to adapt their expectations to ability track placement.

Based on the literature on the immigrant aspiration–achievement paradox, we expected that students with a migration background would be less likely to downwardly adjust their expectations when track placement is below their expectations and more likely to upwardly adjust their expectations when track placement exceeds their expectations. We found little support for this hypothesis. Hence, these findings suggest that the relatively high educational expectations (given academic performance) of students with a migration background are not due to the fact that students with a migration background *respond* differently to tracking signals (cf., Geven, 2019; Pinquart & Ebeling, 2020).

This finding mainly raises questions about the idea that the relatively high expectations among students with a migration background are (partly) due to a lack of knowledge about the educational system. Especially, this idea presupposes that students with a migration background will be less responsive to performance and tracking signals, as they would be less aware of the strong link between track placement and final attainment (cf., Pinquart & Ebeling, 2020). Our findings have fewer implications for other mechanisms that have been proposed to explain the immigrant aspiration–achievement paradox. For example, it is possible that students with a migration background internalize high expectations (from their parents) from an early age on. Even if they adapt their expectations in a similar fashion as students from the native majority, their expectations remain comparably high throughout their educational career. In line with this, previous cross‐sectional research found that the relatively high ambitions of Turkish students in Germany could not be explained by a lack of knowledge about the education system but seemed to be mainly due to students motivation to improve their status (Salikutluk, 2016).

While we found few variations in student responses to track placement by migration background, students from socioeconomically advantaged backgrounds increased their likelihood to expect the Abitur more when their track placement exceeded their initial expectations. Related to this, Forster (2021) showed that parents from socioeconomically advantaged backgrounds were less likely to downwardly adapt their expectations if their child's track placement was below expectations. Forster (2021) argued that high SES parents are more likely to assume that their child will attain college even in light of negative performance signals. They have a greater belief that, with additional effort, educational outcomes can still be improved and are less likely to assume that bad performance is a reflection of a lack of talent. Children may internalize these attitudes.

### 7.1 Limitations

This study also knows some limitations. While our panel design allowed us to shed more light on the causal direction of the relationship between track placement and student expectations, it should be noted that the parallel trends assumption was not fully met. In line with the parallel trends assumption, the expectations of students who were placed in the nonacademic tracks started to diverge from students who were not tracked after track placement took place. However, the expectations of students who were placed in the academic track already started to diverge from that of other students before track placement took place. Hence, changes in their expectations were not (solely) due to track placement itself. Nevertheless, it is important to note that students may receive track placement signals before actual track placement, as students receive a track recommendation from their teacher in third grade. Unfortunately, we did not have information on student expectations prior to third grade.

Relatedly, changes in students' likelihood to expect the Abitur may be due to other factors that change within students over time. German students are not only placed in a specific track after fourth grade but enter an entirely different school with a different student population. Possibly these changes also lead to changes in educational expectations, especially since students may form their expectations in reference to their immediate peer environment.

Finally, we solely relied on German data and focused on the distinction between students with a Western and non‐Western migration background. Hence, we are neither able to capture the (wider) international variation in tracking institutions nor the possible heterogeneity in the experiences of people with different ethnic and/or migration backgrounds. Future research could benefit from cross‐nationally comparative panel data on student expectations that include a larger sample of students with an ethnic minority and/or migration background. In this way, researchers could analyze a wider variety of tracking institutions and make more fine‐grained distinctions between students of different ethnic and/or migration backgrounds. We do want to note that the focus on a single country also has advantages, as different countries tend to vary on a wide range of institutional factors, making it more difficult to shed light on the possible sources of the differences across contexts.

### 7.2 Implications

The finding that German students adapt their educational expectations in response to their track placement is important, as educational expectations are central to educational attainment (Beal & Crockett, 2010; Pinquart & Ebeling, 2020). Since track placements do not always accurately align with students' competencies, students may wrongfully adapt their expectations, thereby potentially affecting future educational chances. That is, research found inaccuracies in teacher track recommendations (Geven et al., 2018; Wang et al., 2018), which causes students to be placed in tracks that do not match their abilities. Hence, track placements may at times become a self‐fulfilling prophecy whereby students internalize false teacher expectations in such a way that they become reality.

The finding that students adapt their expectations according to their school track placement may extend beyond the (German) context of between‐school tracking, as in some comprehensive systems students are tracked *within* schools on a course‐by‐course basis (e.g., the United States and the United Kingdom) (Chmielewski, 2014). In theory, this within‐school tracking (or internal differentiation) is less rigid and characterized by higher levels of track mobility, yet, in practice, track assignment can also have large implications for students' future chances in these systems. Moreover, some comprehensive systems are characterized by high levels of school segregation, which can be seen as an implicit form of tracking (Salchegger, 2016). School segregation causes students to be separated into schools of different quality and this may affect student expectations in a similar vein as between‐school tracking, as students who attend lower‐quality schools will have lower chances to be accepted to (higher quality) University programs. Future studies may want to compare the impact of explicit and implicit, as well as within‐ and between‐school tracking, on student expectations.

Besides the findings that students generally adapt their expectations in response to their track level, our findings also suggest that students from disadvantaged backgrounds are less likely to adjust their expectations upwardly when their track placement exceeds their initial expectations. In this way educational inequalities may be exacerbated, especially since (1) students from disadvantaged backgrounds tend to have lower expectations to start with, even with the same performance levels (Forster et al., 2020) and (2) students from disadvantaged backgrounds tend to receive lower track recommendations from teachers, even with the same performance levels (Geven et al., 2018). It is important to be aware of these potential side effects of track placement. In light of these findings, it may be important for educational professionals to make students aware of the opportunities to still attain an academic qualification after nonacademic track placement.

## CONFLICT OF INTEREST

The authors have no conflict of interest to disclose.

## Data Availability

The data that support the findings of this study are available from NEPS (remote access). Restrictions apply to the availability of these data, which were used under license for this study. Data are available at https://www.neps‐data.de/Data‐Center/Data‐Access (remoteNEPS) with the permission of the Leibniz Institute for Educational Trajectories.

## ENDNOTES

^1^
We also performed analyses in which we distinguished students who are still in primary school from students who attend a school offering multiple tracks (see Table A3 in Appendix). This led to highly similar findings, and the same conclusions as the ones discussed in the text. However, we decided to combine these two groups of students, as the sample sizes in some cells get quite small.

^2^
Due to NEPS restrictions, we are not allowed to directly compare students in different federal states. However, we believe that the difference between students who are tracked and who are not yet tracked is theoretically the most meaningful difference across the Bundesländer given this study's focus.

^3^
We also perform robustness checks in which we consider differences between (1) students from the native majority, (2) students with a Turkish background, (3) students with a background from the FSU or Poland, and (3) students with another migration background.

^4^
In the data, this is a 9‐point scale. More specifically, the following groups were distinguished: 0/1, 2B, 2A, 3A, 3B, 3C, 4A, 5B, 5A, and 6.

^5^
As we are using individual fixed effect models, we cannot estimate the main effect of time‐invariant characteristics, including a student's migration background or parental education. Nevertheless, we are able to estimate differences in expectation change by looking at the interaction terms.

^6^
These calculations are based on the estimates presented in Table 2, Model 1.

## 1

**TABLE A1:** DiD model predicting changes in student expectations distinguishing between native majority, Turkish, FSU/Polish and other migration background

	M2	M3
Grade (ref. = grade 3)		
Grade 5	−0.002	−0.001
	(0.008)	(0.013)
Grade * Match expectation‐track (ref. = Expectation = track)		
Grade 5 * Expectation > track	−0.749***	−0.776***
	(0.037)	(0.045)
Grade 5 * Expectation < track	0.904***	0.855***
	(0.020)	(0.032)
Grade 5 * Not tracked	−0.013	−0.111**
	(0.030)	(0.037)
Grade * Migration background (ref. = Native majority)		
Grade 5 * Turkish	0.020	0.019
	(0.083)	(0.082)
Grade 5 * FSU/Polish	0.003	0.001
	(0.024)	(0.028)
Grade 5 * Other	0.004	0.002
	(0.024)	(0.025)
Grade * Match expectation‐track * Migration background		
Grade 5 * Expectation > track * Turkish	0.096	0.056
	(0.231)	(0.278)
Grade 5 * Expectation > track * FSU/Polish	0.189	0.182
	(0.127)	(0.133)
Grade 5 * Expectation > track * Other	0.085	0.057
	(0.133)	(0.140)
Grade 5 * Expectation < track * Turkish	−0.087	−0.033
	(0.175)	(0.159)
Grade 5 * Expectation < track * FSU/Polish	−0.047	−0.001
	(0.066)	(0.071)
Grade 5 * Expectation < track * Other	−0.087	−0.052
	(0.058)	(0.057)
Grade 5 * Not tracked * Turkish	−0.068	0.023
	(0.250)	(0.261)
Grade 5 * Not tracked * FSU/Polish	0.111	0.162
	(0.110)	(0.104)
Grade 5 * Not tracked * Other	−0.105	−0.059
	(0.112)	(0.117)
Grade * Parental education (ref. = Upper Sec., Voc)		
Grade 5 * ≤ Inter. Sec.		0.015
		(0.059)
Grade 5 * ≥ Bachelor		−0.002
		(0.015)
Grade * Match expectation‐track * Parental education		
Grade 5 * Expectation > track * ≤ Inter. Sec.		0.075
		(0.188)
Grade 5 * Expectation > track * ≥ Bachelor		0.085
		(0.081)
Grade 5 * Expectation < track * ≤ Inter. Sec.		−0.114
		(0.129)
Grade 5 * Expectation < track * ≥ Bachelor		0.089*
		(0.038)
Grade 5 * Not tracked * ≤ Inter. Sec.		−0.089
		(0.157)
Grade 5 * Not tracked * ≥ Bachelor		0.257***
		(0.059)
Constant	0.641***	0.641***
	(0.004)	(0.004)
*N*	5,542	5,542

Note

Data: NEPS SC2, version 8.0.1. Estimates are averaged over 20 imputed data sets. Standard errors in parentheses. All models contain individual‐level fixed‐effects.

* *p* <.05

** *p* <.01

*** *p* <.001.

**TABLE A2:** DiD model predicting changes in student expectations (track placement)

	M1	M2	M3
Grade (ref. = grade 3)			
Grade 4	−0.061	−0.057	−0.046
	(0.034)	(0.039)	(0.042)
Grade 5	−0.154***	−0.164***	−0.175***
	(0.029)	(0.033)	(0.035)
Grade 6	−0.164***	−0.170***	−0.186***
	(0.028)	(0.031)	(0.034)
Grade * Track (ref. = Vocational track)			
Grade 4 * Academic track	0.169***	0.163***	0.155**
	(0.036)	(0.041)	(0.049)
Grade 4 * Not tracked	0.067	0.064	0.006
	(0.043)	(0.048)	(0.057)
Grade 5 * Academic track	0.333***	0.344***	0.355***
	(0.030)	(0.035)	(0.039)
Grade 5 * Not tracked	0.137**	0.146**	0.063
	(0.040)	(0.045)	(0.051)
Grade 6 * Academic track	0.325***	0.325***	0.338***
	(0.030)	(0.033)	(0.039)
Grade 6 * Not tracked	0.177***	0.186***	0.114**
	(0.040)	(0.044)	(0.050)
Grade * Migration background (ref. = Native majority)			
Grade 4 * Non‐Western		0.020	0.086
		(0.147)	(0.152)
Grade 4 * Western		−0.035	0.024
		(0.086)	(0.107)
Grade 5 * Non‐Western		0.050	0.081
		(0.160)	(0.156)
Grade 5 * Western		0.047	0.071
		(0.081)	(0.094)
Grade 6 * Non‐Western		0.123	0.160
		(0.156)	(0.155)
Grade 6 * Western		−0.002	0.026
		(0.084)	(0.099)
Grade * Track * Migration background			
Grade 4 * Academic track * Non‐Western		−0.001	−0.080
		(0.158)	(0.161)
Grade 4 * Academic track * Western		0.046	−0.030
		(0.096)	(0.118)
Grade 4 * Not tracked * Non‐Western		−0.183	−0.224
		(0.188)	(0.199)
Grade 4 * Not tracked * Western		0.104	0.066
		(0.142)	(0.154)
Grade 5 * Academic track * Non‐Western		−0.077	−0.112
		(0.170)	(0.164)
Grade 5 * Academic track * Western		−0.046	−0.074
		(0.086)	(0.100)
Grade 5 * Not tracked * Non‐Western		−0.183	−0.169
		(0.216)	(0.216)
Grade 5 * Not tracked * Western		0.035	0.055
		(0.123)	(0.129)
Grade 6 * Academic track * Non‐Western		−0.085	−0.131
		(0.167)	(0.165)
Grade 6 * Academic tracked * Western		0.039	−0.003
		(0.091)	(0.105)
Grade 6 * Not tracked * Non‐Western		−0.389	−0.418
		(0.220)	(0.229)
Grade 6 * Not tracked * Western		0.087	0.089
		(0.126)	(0.132)
Grade * Parental education (ref. = Upper Sec., Voc)			
Grade 4 * ≤ Inter. Sec.			−0.210
			(0.146)
Grade 4 * ≥ Bachelor			−0.038
			(0.077)
Grade 5 * ≤ Inter. Sec.			−0.077
			(0.142)
Grade 5 * ≥ Bachelor			0.048
			(0.078)
Grade 6 * ≤ Inter. Sec.			−0.089
			(0.161)
Grade 6 * ≥ Bachelor			0.065
			(0.084)
Grade * Track * Parental education			
Grade 4 * Academic track * ≤ Inter. Sec.			0.328
			(0.175)
Grade 4 * Academic track * ≥ Bachelor			0.032
			(0.085)
Grade 4 * Not tracked * ≤ Inter. Sec.			0.174
			(0.217)
Grade 4 * Not tracked * ≥ Bachelor			0.164
			(0.097)
Grade 5 * Academic track * ≤ Inter. Sec.			0.113
			(0.173)
Grade 5 * Academic track * ≥ Bachelor			−0.049
			(0.083)
Grade 5 * Not tracked * ≤ Inter. Sec.			0.000
			(0.209)
Grade 5 * Not tracked * ≥ Bachelor			0.206
			(0.099)
Grade 6 * Academic track * ≤ Inter. Sec.			0.190
			(0.185)
Grade 6 * Academic track * ≥ Bachelor			−0.061
			(0.089)
Grade 6 * Not tracked * ≤ Inter. Sec.			0.105
			(0.225)
Grade 6 * Not tracked * ≥ Bachelor			0.166
			(0.010)
Constant	0.646***	0.646***	0.646***
	(0.007)	(0.007)	(0.007)
*N*	10,236	10,236	10,236

Note

Data: NEPS SC2, version 8.0.1. Estimates are averaged over 20 imputed data sets. Standard errors in parentheses. All models contain individual‐level fixed‐effects.

* *p* < .05

** *p* < .01

*** *p* < .001.

**TABLE A3:** DiD model predicting changes in student expectations differentiating between students in primary school and combined tracks

	M1	M2	M3
Grade (ref. = grade 3)			
Grade 5	−0.001	−0.002	−0.003
	(0.007)	(0.008)	(0.008)
Grade * Match expectation‐track (ref. = Expectation = track)			
Grade 5 * Expectation > track	−0.717***	−0.752***	−0.746***
	(0.033)	(0.036)	(0.038)
Grade 5 * Expectation < track	0.887***	0.904***	0.899***
	(0.018)	(0.019)	(0.021)
Grade 5 * Combi track	−0.031	−0.030	−0.035
	(0.033)	(0.035)	(0.036)
Grade 5 * Primary school	0.035	0.033	0.028
	(0.048)	(0.050)	(0.052)
Grade * Migration background (ref. = Native majority)			
Grade 5 * Non‐Western		0.034	0.028
		(0.038)	(0.038)
Grade 5 * Western		−0.004	−0.009
		(0.023)	(0.024)
Grade * Match expectation‐track * Migration background			
Grade 5 * Expectation > track * Non‐Western		−0.023	0.063
		(0.136)	(0.189)
Grade 5 * Expectation > track * Western		0.228*	0.249*
		(0.112)	(0.115)
Grade 5 * Expectation < track * Non‐Western		−0.193*	−0.128
		(0.094)	(0.087)
Grade 5 * Expectation < track * Western		−0.040	−0.011
		(0.055)	(0.055)
Grade 5 * Combi track * Non‐Western		−0.143	−0.121
		(0.185)	(0.188)
Grade 5 * Combi track * Western		0.052	0.068
		(0.103)	(0.102)
Grade 5 * Primary school * Non‐Western		−0.232	−0.184
		(0.219)	(0.219)
Grade 5 * Primary school * Western		−0.147	0.148
		(0.177)	(0.173)
Grade * Parental education (ref. = Upper Sec., Voc)			
Grade 5 * ≤ Inter. Sec.			0.090
			(0.080)
Grade 5 * ≥ Bachelor			0.002
			(0.018)
Grade * Match expectation‐track * Parental education			
Grade 5 * Expectation > track * ≤ Inter. Sec.			−0.210
			(0.228)
Grade 5 * Expectation > track * ≥ Bachelor			−0.166
			(0.132)
Grade 5 * Expectation < track * ≤ Inter. Sec.			−0.496*
			(0.201)
Grade 5 * Expectation < track * ≥ Bachelor			0.073
			(0.040)
Grade 5 * Combi track * ≤ Inter. Sec.			−0.190
			(0.253)
Grade 5 * Combi track * ≥ Bachelor			0.159
			(0.114)
Grade 5 * Primary school * ≤ Inter. Sec.			−0.175
			(0.225)
Grade 5 * Primary school * ≥ Bachelor			0.419
			(0.252)
Constant	0.641***	0.641***	0.641***
	(0.004)	(0.004)	(0.004)
*N*	5,542	5,542	5,542

Note

Data: NEPS SC2, version 8.0.1. Estimates are averaged over 20 imputed data sets. Standard errors in parentheses. All models contain individual‐level fixed‐effects.

* *p* < .05

** *p* < .01

*** *p* < .001.
